# The impact of the COVID-19 outbreak on the medico-legal and human rights of psychiatric patients

**DOI:** 10.1192/j.eurpsy.2020.58

**Published:** 2020-05-29

**Authors:** Johannes Thome, Andrew N. Coogan, Frederick Simon, Matthias Fischer, Oliver Tucha, Frank Faltraco, Donatella Marazziti, Hermann Butzer

**Affiliations:** 1 Clinic and Policlinic for Psychiatry and Psychotherapy, University of Rostock, Rostock, Germany; 2 Department of Psychology, Maynooth University, National University of Ireland, Maynooth, Ireland; 3 Department of Clinical and Developmental Neuropsychology, University of Groningen, Groningen, The Netherlands; 4 Department of Experimental and Clinical Medicine, Section of Psychiatry, University of Pisa, Pisa, Italy; 5 UniCamillus University of Rome, Brain Research Foundation, Lucca, Italy; 6 Law School, Leibniz University Hannover, Hannover, Germany

## Abstract

The COVID-19 pandemic has raised significant concerns for population mental health and the effective provision of mental health services in the light of increased demands and barriers to service delivery [1]. Particular attention is being directed toward the possible neuropsychiatric sequelae of both COVID-19 and of the stringent societal mitigation steps deployed by national governments, concerns that are informed by historical increases in the incidence of psychotic disorders following influenza pandemics [2]. However, so far there has been scant attention paid to other important areas of psychiatry during COVID-19, including medico-legal aspects and human rights. In this paper, we discuss the legal implications for psychiatry of the COVID-19 pandemic and report a novel situation in which psychiatric patients may experience diminution of their statutory protections. We believe that this represents a paradigm shift in psychiatric care and that the consideration of the fundamental rights of psychiatric patients as “less important” than infection control measures compel mental health professionals to “advocate for … patients and their caregivers” in this time of crisis [1].

The COVID-19 pandemic has raised significant concerns for population mental health and the effective provision of mental health services in the light of increased demands and barriers to service delivery [1]. Particular attention is being directed towards the possible neuropsychiatric sequelae of both COVID-19 and of the stringent societal mitigation steps deployed by national governments, concerns that are informed by historical increases in the incidence of psychotic disorders following influenza pandemics [2]. However, so far there has been scant attention paid to other important areas of psychiatry during COVID-19, including medico-legal aspects and human rights. In this paper, we discuss the legal implications for psychiatry of the COVID-19 pandemic and report a novel situation in which psychiatric patients may experience diminution of their statutory protections. We believe that this represents a paradigm shift in psychiatric care and that the consideration of the fundamental rights of psychiatric patients as “less important” than infection control measures compels mental health professionals to “advocate for … patients and their caregivers” in this time of crisis [1].

Coercion and restraint are highly problematic and controversial interventions in the treatment of patients suffering from severe mental disorders. In light of the legitimate concerns regarding the potential for the violation of civil and human rights [3], many countries have established strict legal procedures in order to ensure that patient rights are safeguarded. This process is often under independent judicial control; however, this important layer of protection is now under threat. Severe pandemics, such as the present COVID-19 outbreak, lead to profound societal changes, with the potential to alter medico-legal frameworks of impacted countries. In many countries during the COVID-19 pandemic, coercive powers have been legally extended to healthcare professionals and/or other public servants who can compel members of the public into isolation, quarantine, and treatment [4]. These emergency powers can also affect those who are already being involuntarily treated, such as psychiatric patients. According to German law, every patient who is compulsorily admitted to a psychiatric clinic, or physically restrained, must have their case legally reviewed and be offered the opportunity of a personal hearing with a judge. Without the approval by a judge, such measures are illegal and cannot be taken.

In the wake of the COVID-19 outbreak, all public institutions, including psychiatric clinics and district courts, are under immense pressure and strain, struggling to maintain even the most basic services [1]. As in other parts of the world, exceptional and nationwide measures were taken in Germany to prevent the rapid spread of the virus with some of these measures directly affecting individuals suffering from severe psychiatric conditions. According to German law, it is mandatory that psychiatric patients undergoing coercion (involuntary admission to a closed ward) and/or restraint (fixation) be seen by an independent judge. Only, after a personal hearing and a legal verdict by this judge, mental health professionals are allowed to proceed with coercive measures. However, during the COVID-19 outbreak, our clinic was informed, without prior consultation or notice, by the district court that judges’ visits to acute psychiatric wards in order to review patients under restraint or coercive measures would be discontinued, and that effectively personal hearings would be suspended. Instead, judges would make legal decisions without any personal hearing, based on documents submitted to the court by psychiatrists. The motivation was the “protection of medical and legal staff, as well as patients.” From our perspective as mental health professionals, we believe that this procedure is highly problematic, as our patients are bereft of a fundamental right that legally limits the scope of psychiatric intervention. In addition, it severely interferes with patients’ personal and human rights under both German and European law. From a legal perspective, this position has been both defended [5] and criticized [6] by experts. It has been argued that the protection of judges from infectious diseases not only justifies the discontinuation of legally guaranteed personal hearings of patients who undergo the process of psychiatric treatment against their will, but that this disruption is mandatory given the unique circumstances of the present situation [5]. In contrast, it has also been opined that the judicial hearing represents an integral part of the constitutional rights of psychiatric patients and that such hearings cannot be withdrawn. In addition, the risk of infection can be considerably reduced by using protective measures or electronic means of communication [6].

This dilemma clearly illustrates the fundamental legal and ethical questions of the current COVID-19 pandemic beyond strictly medical issues. The need for the so-called “pandethics” has already been discussed in the context of previous influenza pandemics, and several major ethical issues had been identified [7]. Although these issues included important topics, such as the obligation of individuals to avoid infecting others, the duty to treat, the allocation of scarce resources, and coercive physical distancing measures [7], the specific ethical implications for the vulnerable group of psychiatric patients were not explicitly considered. While some authors have emphasized important general principles such as legitimacy, necessity, effectiveness, proportionality, and fairness during periods of pandemic [4], there has not been particular focus on the characteristic needs of specific vulnerable groups, such as patients with mental disorders. In the wake of the SARS-CoV-1, it was suggested that ethical issues related to the pandemic require a dynamic framework that is constantly re-evaluated and refined according to the latest available information [8]. The development of such ethical frameworks should undergo a “stakeholder engagement process” and the results should become central to future planning [8]. This raises the question of how these fundamental values and principles, such as the “respect for individual rights” and the “protection of individuals” [4], can be translated into the specific medico-legal framework of psychiatry and mental health service provision. Based on ideas developed by Daniels [9], five key values in the ethical process have been identified [8]: Accountability, Inclusiveness, Openness and Transparency, Reasonableness, and Responsiveness.

Considering these value-based principles, practical advices for mental health workers on how to protect and defend the rights of patients with severe mental illness in the circumstances of a pandemic may include: (a) close links being built and maintained with nonmedical professionals (such as those within the legal sector) involved in psychiatric patient care; (b) regular contact with external agents being maintained to ensure that no unilateral decisions are taken without prior consultation with mental health experts, representatives of patients, and their relatives; (c) expert information about the medical (and not just psychiatric) background of patients being communicated to these external agents with a special emphasis on the presence of infectious disease and on possible preventive measures; (d) the use of electronic means of communication being used as a possible route to guaranteeing patients’ access to their fundamental rights while simultaneously protecting the health of their legal representatives; and (e) awareness being created for the special needs of vulnerable groups (such as psychiatric patients) by mental health professionals in collaboration with decision-makers in social services and political and legal institutions. By following these guidelines, mental health professionals working in psychiatric clinics can significantly contribute to guarantee the highest ethical and clinical standards for their patients.

It is clear that the COVID-19 pandemic evokes anxieties and fears in public servants, including judges, and that this can impact on the normal functioning of essential legal services. Additionally, there is a risk that one of the most vulnerable groups in society, patients with severe mental disorders who already have limited public agency, are at risk of being most negatively affected. Therefore, it can be concluded that medical and psychiatric staff play an important role in the education of nonmedical officials (such as judges) of the potential risks of viral infection and that they also have a role in providing effective protective equipment and procedures to this group of professionals to facilitate them in fulfilling their vital legal responsibilities with regard to the safeguarding of human rights of psychiatric patients. By adhering to standard hygiene practice and to the specific guidelines published by health authorities, such as the European Centre for Disease Prevention and Control, certain essential services could be maintained even in the context of extraordinary measures being taken in order to contain the pandemic. The use of remote, online facilities could further help to ensure that at least a minimum level of external care can continue in the psychiatric and medico-legal sectors [10]. More than ever before, psychiatrists need to protect and defend the fundamental rights of their patients now.
